# Homicides Disguised as Fire Deaths

**DOI:** 10.15388/Amed.2023.30.1.10

**Published:** 2023-05-16

**Authors:** Gabrielė Žiūkaitė, Marta Jasaitė, Sigitas Chmieliauskas, Diana Vasiljevaitė, Sigitas Laima, Dalius Banionis, Jurgita Stasiūnienė

**Affiliations:** Faculty of Medicine, Vilnius University, Vilnius, Lithuania; Faculty of Medicine, Vilnius University, Vilnius, Lithuania; Department of Pathology, Forensic Medicine, Institute of Biomedical Sciences of the Faculty of Medicine of Vilnius University, Vilnius, Lithuania; Department of Pathology, Forensic Medicine, Institute of Biomedical Sciences of the Faculty of Medicine of Vilnius University, Vilnius, Lithuania.; Department of Pathology, Forensic Medicine, Institute of Biomedical Sciences of the Faculty of Medicine of Vilnius University; Department of Pathology, Forensic Medicine, Institute of Biomedical Sciences of the Faculty of Medicine of Vilnius University, Vilnius, Lithuania; Department of Pathology, Forensic Medicine, Institute of Biomedical Sciences of the Faculty of Medicine of Vilnius University, Vilnius, Lithuania

**Keywords:** forensic science, homicide, burned bodies, antemortem burning, postmortem burning

## Abstract

**Background:**

When conducting a forensic examination of burnt bodies, it is important to determine whether the victim was exposed to fire while alive or after death. The differential diagnosis between antemortem and postmortem burning is difficult and often cannot be made based on information obtained solely from the autopsy. The aim of the study is to review current literature on this topic and present clinical cases that illustrate how challenging the determination of vitality during the fire and manner of death can be.

**Materials and methods:**

We present four cases of burnt homicide victims, illustrating the complexity of forensic determination of the cause of death in the fire and the importance of differential diagnosis of antemortem and postmortem exposure to flames.

**Results:**

In the forensic assessment autopsy is a fundamental to determine the cause of death. When death is related to fire, particular findings during autopsy can help to suspect that the victim was alive. One of the main antemortem signs is the deposition of soot in the respiratory tract. Another important test is the toxicological analysis, which determines the level of carboxyhaemoglobin in the blood: a concentration of more than 50% indicates that the person died in the fire.

**Conclusions:**

Forensic examination of burnt bodies requires a comprehensive and detailed assessment of all available data. The autopsy, together with additional diagnostic forensic methods, including histological examination, toxicological analysis and postmortem computed tomography, allows the exact cause of death to be determined.

## Santrauka.

**Įvadas:**

Atliekant apdegusių kūnų teismo medicininę ekspertizę, svarbu nustatyti, nukentėjusysis buvo veikiamas aukštos temperatūros ir atviros liepsnos būdamas gyvas ar po mirties. Diferencinė priešmirtinio ir pomirtinio aukštos temperatūros poveikio diagnostika yra sudėtinga ir kartais remiantis vien tik autopsijos duomenimis negali būti vertinama. Mūsų tyrimo tikslas buvo apžvelgti naujausią literatūrą šia tema ir pateikti klinikinių atvejų, kurie iliustruoja, kiek sudėtinga gali būti nustatyti apdegusių kūnų mirties priežastį.

**Medžiaga ir metodai:**

Pateikiame keturių sudegintų žmogžudysčių aukų atvejus, iliustruojančius, koks sudėtingas gali būti teismo medicininis mirties priežasties nustatymas, kai yra aukštos temperatūros ir atviros liepsnos poveikis, bei kokia svarbi diferencinė priešmirtinio ir pomirtinio aukštos temperatūros poveikio diagnostika.

**Rezultatai:**

Atliekant mirusiųjų teismo medicininį tyrimą, autopsija yra esminė dalis norint nustatyti mirties priežastį. Kai mirtis yra susijusi su aukštos temperatūros poveikiu, autopsijos metu konkretūs radiniai gali nurodyti, kad auka buvo gyva, kai kūnas buvo veikiamas aukštos temperatūros ar atviros liepsnos. Vienas iš priešmirtinių požymių – suodžiai kvėpavimo takuose. Kitas svarbus tyrimas – toksikologinis, kurio metu nustatomas karboksihemoglobino kiekis kraujyje: jo koncentracija, didesnė nei 50 proc., rodo, kad asmuo mirė gaisro metu.

**Išvada:**

Atliekant apdegusių kūnų teismo medicininę ekspertizę, reikia atlikti kompleksinį ir detalų visų turimų duomenų vertinimą. Atlikta autopsija su papildomais diagnostiniais teismo medicininiais tyrimo metodais, įskaitant histologinį tyrimą, toksikologinę analizę, pomirtinę kompiuterinę tomografiją, leidžia nustatyti tikslią mirties priežastį.

## Introduction

During the forensic examination of burnt bodies, the question of whether the victim was exposed to fire before or after death is the major concern. Differentiation between antemortem and postmortem burns is an important issue in these cases, and it may even be impossible to do this, especially in charred bodies. The combination of diagnostic methods should be used, and the diagnosis should not be based only on the autopsy findings. We review current literature and report 4 cases of burnt victims that illustrate how challenging the determination of vitality during the fire and manner of death can be.

## Methods

### Study design and data source

The performed retrospective study included 4 victims, whose cause of death was homicide with the burning of the body after death. The data, regarding the postmortem investigation of the victims, was obtained from the Lithuanian State Forensic Medicine Service database. All decedents received full autopsies. In every case, there was information provided by the law enforcement agencies, including scene of the incident, time of death, and the presumable death mechanism.

### Identification of cases

The study involved 4 cases, where the cause of death was homicide with a cover-up of the crime by burning the victims. All the victims died suddenly without receiving any medical treatment. Cases, where the cause of death due to body charring could not be determined, were excluded from the analysis. The circumstances of all the cases were determined as a homicide.

### Limitations

No postmortem computed tomography (PMCT) scans were performed on the homicide victims.

### Histological methods

Histological sections were cut and prepared for routine light microscopy. Histomorphological features of the clot were examined using hematoxylin and eosin (H&E) staining. Perl’s Prussian blue reaction was used to detect ferric iron and Masson’s Trichrome staining for collagen fibers. The H&E staining consists of several stages: removal of paraffin, staining, and dehydration. Sections are deparaffinated by keeping them sequentially in absolute alcohol, 96% and 70% ethanol, and distilled water for a certain time. After that specimens are stained with hematoxylin solution and then continuously irrigated with flowing water. Afterward, an eosin-floxin solution is applied. Finally, specimens are quickly sequentially dehydrated in 70%, 90%, and absolute alcohol and enclosed with covering material. The nucleus and other DNA/RNA-containing structures are dyed in blue-violet color while the cytoplasm and matrix in different pink tints.

### Evaluation of the methods used for carboxyhemoglobin analysis in postmortem blood

Automated spectrophotometers, called CO-oximeters, were used which perform differential spectrophotometric measurements on blood samples. These CO-oximeters require little or no sample preparation and can measure total hemoglobin (Hb), carboxyhemoglobin (COHb), and other forms of hemoglobin simultaneously.

### Characteristics of burn lesions

The fire starts by burning off the skin and the soft tissues resulting in skeletal muscle exposure. Exposed muscles then contract and shrink because of the heat, leading to a flexion deformation of the limbs. The upper limbs present a characteristic flexion the body takes the position of a boxer. Such thermal consequences on the burned body’s overall state appear quickly after exposure to the fire.

### Antemortem burns

Histopathological findings of burnt skin can show cleft formation at the junction of epithelium and the connective tissue or separation of the epidermis from the dermis and breaking of the epithelium; separation up to basal and granular cell layer or cellular detail being totally disorganized; homogenization and vacuolization of connective tissue; petechial haemorrhages; epithelial cells being flattened and elongated; capillary dilatation; edema and margination of leucocytes; congestion and infiltration.

### Postmortem burns

Histopathological screening of the charred skin fragments showed that the outer crisp surface was completely destroyed macroscopically. Heat-related fluid shifts may cause vesicular detachment of the epidermis (false burn blisters) on the skin. But frequently underneath there was a thin layer of dehydrated skin, which showed moderate preservation of cellular patterns. No inflammatory reaction with polymorphonuclear leucocytic infiltration. The dermis, subcutaneous adipose, and muscle tissues were not always visible during the autopsy. They can range from minor, local, superficial burns of the skin to charred, fragile and dry skeletal remains.

## Practical examples

### Case 1

**Fig. 1.**
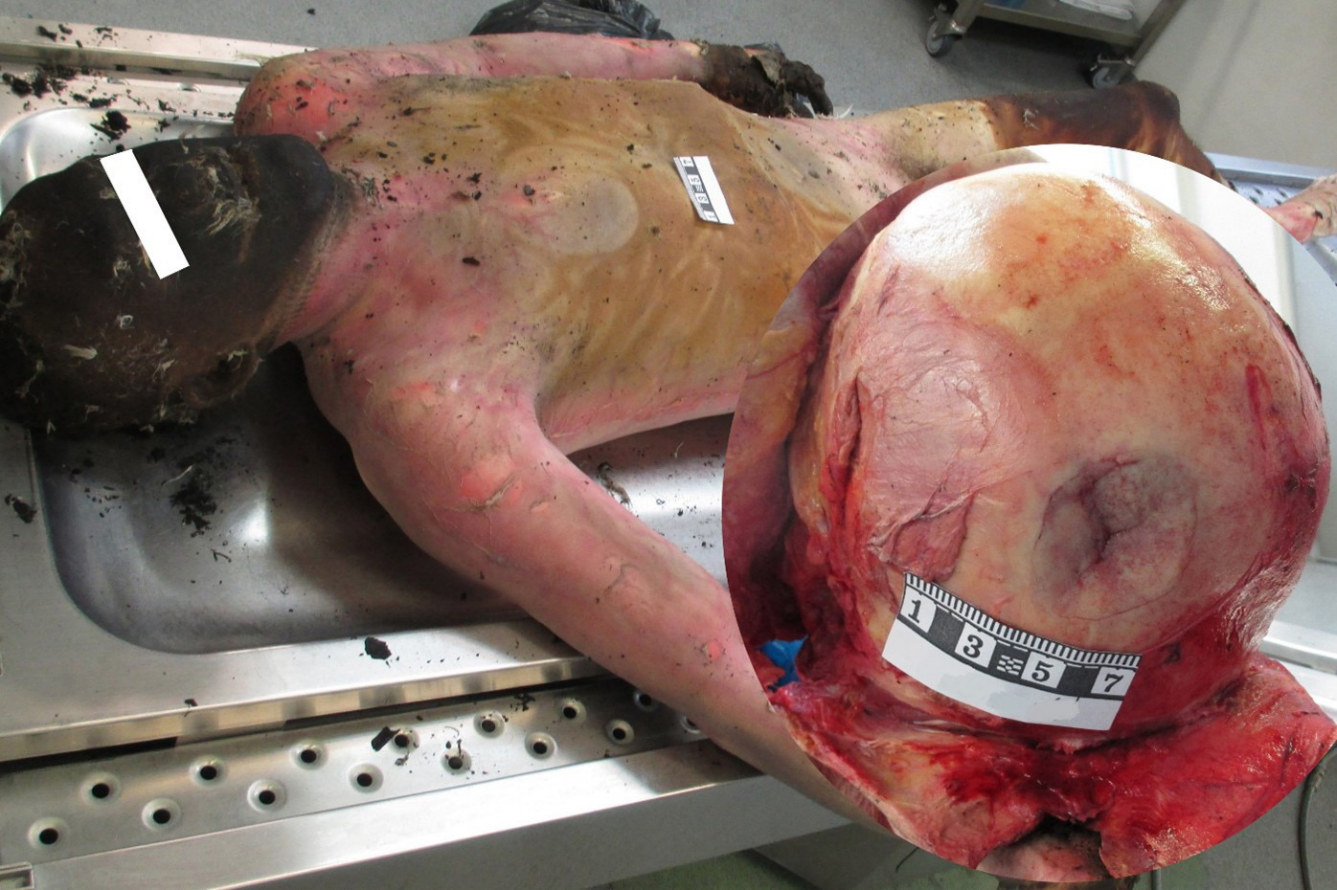
A compression fracture of the left temporal bone

The burnt body of a 53-year-old man was found at the fire scene. All layers of the skin are observed to be damaged in the areas of the body not covered by clothes, charred soft tissues and muscles without a pronounced tissue reaction. Histological examination revealed no inflammatory reaction of tissue. I°-IV° postmortem burns cover about 45% of total body surface area (TBSA). A compression fracture of the left temporal bone was found with hemorrhage of the surrounding soft tissues, infiltrating hemorrhages in the dura mater, isolated hemorrhages in the left temporal lobe (Fig. 1).

A projection of the fracture shows a compression of the brain, replicating a compression fracture. Based on the macro- and microscopic view, head injury was caused by a hard, blunt object not long before death. Lividity is observed in cherry-red discoloration of skin, soft tissue, and internal organs in a shade of light pink. Furthermore, traces of soot on the surface of the tongue, vocal folds, trachea, large bronchi, reddened tracheal mucosa were found. Blood was taken from the iliac veins for chemical testing for toxic, narcotic and potent substances, alcohol and CO-Hb. In addition, for alcohol analysis urine sample was taken. According to toxicology analyses 3.07‰ ethyl alcohol, 58.8% CO-Hb concentration was found in the blood and 3.96‰ ethyl alcohol concentration in urine. No amphetamines, amitriptyline, barbiturates, benzodiazepines, diphenhydramine, ephedrine, phenothiazines, cocaine, metabolites of cocaine, methamphetamisole, opiates, tetrahydrocannabinol, metabolites of tetrahydrocannabinol, were detected in the blood. Overall, in spite of the fact that the absence of tissue reaction is a postmortem sign, majority of the findings (for example, CO-Hb > 50%, cherry-red lividity, soot deposition in trachea, large bronchi) prove that the cause of death is exposure to toxic effect of carbon monoxide (CO) and combustion products.

### Case 2

**Fig. 2.**
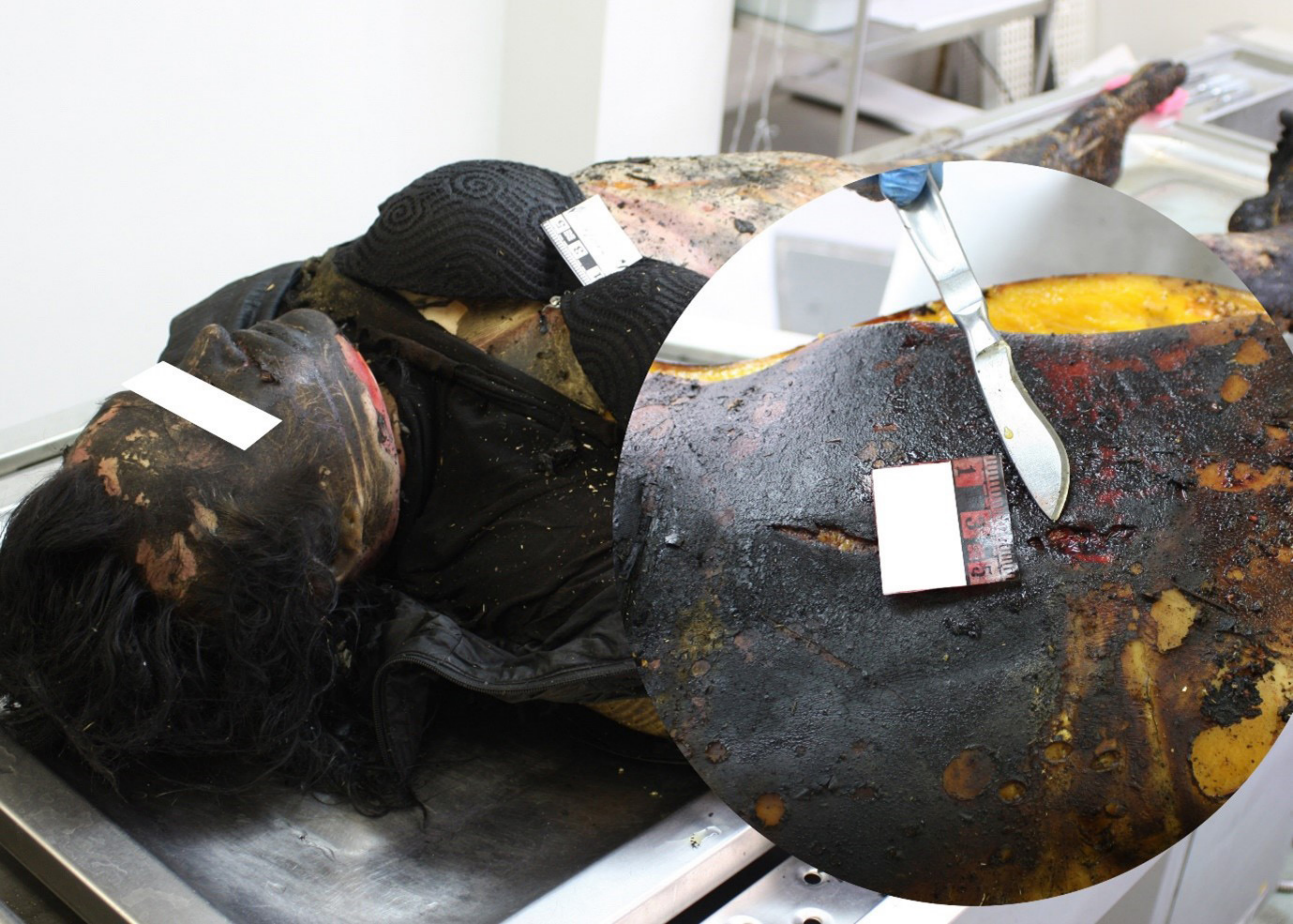
Multiple stabbing-cutting injuries to the chest

The burnt body of a 60-year-old woman was found at the fire site. During investigation the remains of burnt clothes, burning and charring of the whole body are observed. Due to charring of the body, lividity is not distinguished. Histological examination revealed no inflammatory reaction of tissue. Multiple stabbing-cutting injuries to the chest with pulmonary lesions, inflicted by a flat, single-edged tool(s) with stabbing-cutting properties, shortly before death were found (Fig. 2).

No soot was detected in the respiratory tract. Blood was taken from the iliac veins for chemical testing for toxic, narcotic and potent substances, alcohol and CO-Hb. Toxicological analysis showed no amphetamines, amitriptyline, barbiturates, benzodiazepines, diphenhydramine, ephedrine, phenothiazines, cocaine, metabolites of cocaine, methamphetamisole, opiates, tetrahydrocannabinol, metabolites of tetrahydrocannabinol, alcohol and CO-Hb in the blood. Due to the postmortem burning and charring of the whole body, it is impossible to say whether the body of the victim started burning while she was alive or dead. According to the toxicological data, it is likely that the victim was no longer breathing in an environment where fire was burning, and CO was formed. The cause of death is acute respiratory function and heart failure due to stabbing-cutting injuries of the chest.

### Case 3

**Fig. 3.**
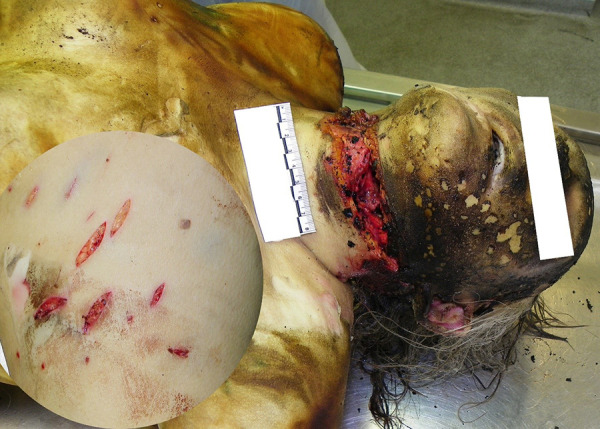
Multiple stabbing-cutting and cutting injuries

**Fig. 4.**
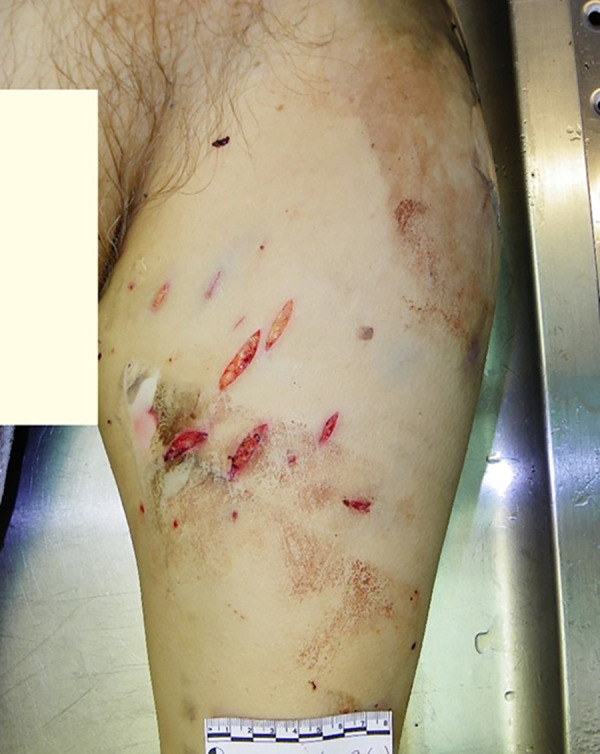
Multiple stabbing-cutting and cutting injuries

A burnt corpse of an 86-year-old female was found in the forest. Multiple stabbing-cutting and cutting injuries were found: a slash wound to the neck with incisions in the hyoid bone, esophagus and left external carotid artery and a complete cut of the left superior thyroid artery (Figs. 3, 4).

During examination of the body, multiple stab wounds to the pubic area and both thighs with extensive soft tissue bruising and a fully severed left femoral vein were observed. IIB-III° burns covering about 43% of TBSA caused by an open flame, presumably after death, as there is no fluid filled epidermal blisters. Histological examination revealed no inflammatory reaction of tissue. On the dorsal surface of the body, there are single, pink-colored lividity of weak intensity, which do not change color when pressed with a finger. Blood was taken from the iliac veins for chemical testing for alcohol, CO-Hb, amphetamines, amitriptyline, barbiturates, benzodiazepines, diphenhydramine, ephedrine, phenothiazines, cocaine, metabolites of cocaine, methamphetamisole, opiates, tetrahydrocannabinol and tetrahydrocannabinol. Toxic, narcotic, potent substances, alcohol and CO-Hb were not discovered by toxicological analysis in the blood. Furthermore, there was no swelling or soot in the respiratory system which supports hypothesis that fire started after the victim was deceased, therefore the main cause of death is acute external bleeding caused by multiple stabbing-cutting and cutting injuries.

### Case 4

**Fig. 5.**
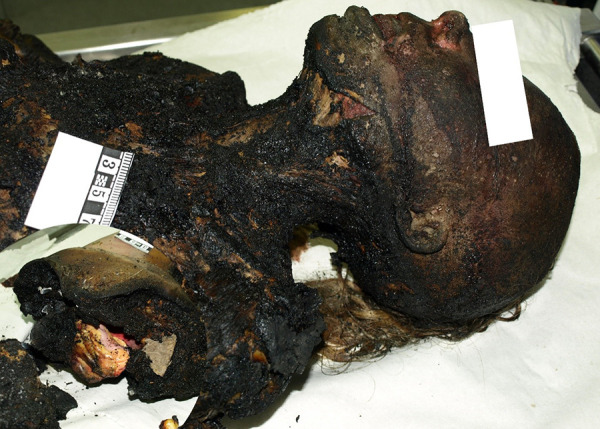
The body is completely split (burnt) in the middle part of the body

A burnt body of a 40-year-old woman was found in the forest. The body is completely split (burnt) in the middle part of the body (Fig. 5). The upper part of the body ends at the 10th thoracic vertebra and the remaining lower part of body begins in the front just above the navel, on the left at the level of the lower quadrant of the buttock, on the right at the right hip joint. Burns cover about 90% of TBSA, complete burning of the soft tissues of the torso, hands, and bones. These burns are postmortem, as indicated by the lack of reaction of the surrounding tissues during the examination of the body and the data of the histological examination. Histological examination revealed no inflammatory reaction of tissue. Moreover, bruises were found in the soft tissues of the tongue, face, and both temporal areas without any cellular reaction. Observed injuries were caused by contact with hard blunt objects and it is probable that they could have been caused by blows to the above-mentioned areas of the face. Nonetheless, these hemorrhages are nonfatal injuries. On the back surface of the left thigh, light purple lividity can be distinguished, which does not change color when pressed with a finger.

Examining the respiratory tract reveals greyish-yellow mucus with absence of soot. Blood was taken from the arterial blood vessels of the legs for chemical analysis for the detection of alcohol and narcotic (opiates and nicotine) substances. Toxicological examination revealed 3.26‰ ethyl alcohol concentration in blood, no narcotic substances were found. However, the concentration of CO-Hb was not established. Overall, it is impossible to ascertain the cause of death due to the corpse’s intense scorching and charring, but the evidence we have collected enables us to conclude that the body’s thermal burn occurred postmortem. Based on the details provided by the police, it was revealed that the victim was strangled and then burned.

The obtained data regarding characteristics of cases and findings are summarized in Table 1.

**Table 1.** Characteristics of cases and findings of investigations

	**Case 1**	**Case 2**	**Case 3**	**Case 4**
**Sex**	Male	Female	Female	Female
**Age**	53	60	86	40
**Autopsy findings**	Burnings of TBSA: 45% Compression fracture of the left temporal bone Traces of soot in the respiratory tract Absence of tissue reaction Cherry-red lividity	Multiple stabbing-cutting injuries to the chest with pulmonary lesions No soot in the respiratory tract Absence of fluid filled epidermal blisters	Burnings of TBSA: 43% Slash wound to the neck, complete cut of the left superior thyroid artery Stab wounds to the pubic area and both thighs, fully severed left femoral vein No soot in the respiratory tract Pink-colored lividity	Burnings of TBSA: 90% Nonfatal injuries on the face No soot in the respiratory tract Absence of tissue reaction Light purple lividity
**Histological findings**	no inflammatory reaction	no inflammatory reaction	no inflammatory reaction	no inflammatory reaction
**Toxicology**	58.8% CO-Hb 3.07‰ ethyl alcohol. Toxic, narcotic, potent substances not found	0% CO-Hb 0‰ ethyl alcohol. Toxic, narcotic, potent substances not found	0% CO-Hb 0‰ ethyl alcohol. Toxic, narcotic, potent substances not found	0% CO-Hb 3.26‰ ethyl alcohol. Toxic, narcotic, potent substances not found
**Cause of death and conclusion**	Exposure to toxic effect of CO and combustion products Postmortem burning	Acute respiratory function and heart failure due to stabbing-cutting injuries of the chest Postmortem burning	Acute external bleeding caused by multiple stabbing-cutting and cutting injuries Postmortem burning	Asphyxia Postmortem burning

TBSA – total body surface area; CO-Hb – carboxyhemoglobin; CO – carbon monoxide

## Discussion

There is currently no exact indicator to distinguish between antemortem burns and postmortem burns. However, there are few criteria that could indicate whether the victim was alive or not when the fire broke out. The data from reviewing the literature concerning valuable parameters that may help to differentiate between antemortem and postmortem burning are summarized in Table 2.

**Table 2.** Specific features of antemortem and postmortem burning

**Features**	**Antemortem**	**Postmortem**
*Toxicological analysis (4,7–11)*	Lethal dose of CO-Hb >50%* *In case of severe CVD 10–30%	CO-Hb < 50%
*Lividity (11)*	Cherry-red	Bluish-purple
*Soot, thermal burns of organs (4–7)*	Soot in the respiratory tract Thermal burns in the airways	Absent
*Heat-hematoma or epidural hematoma caused by trauma (4,15)*	CO-Hb concentration in hematoma blood is similar to peripheral blood; PMCT: low density, crescent shaped, crossing the midline, detaching the venous sinus	CO-Hb is absent or less than peripheral blood; PMCT: dense, convex and lens shaped
*Fractures (13,14)*	May include deeper body areas Bilateral and symmetrical involvement Longitudinal trans-diploic fractures	Superficial areas Usually asymmetrical Perpendicular disruption to the bone surface
*Burns (1–3)*	Line of redness around the burn area; Blisters: red base, full of serous fluid; Histopathological examination: small areas of hemorrhages, PMN infiltration	No line of redness; Blisters (if present): pale, yellow without a red base, filled with air and clear fluid; Histopathological examination: no characteristic changes

CO-Hb – carboxyhemoglobin; CVD – cardiovascular disease; PMN – polymorphonuclear

In the forensic assessment autopsy is a fundamental to determine the cause of death. When death is related to fire, particular findings during autopsy can help to suspect that the victim was alive. External signs that the body was exposed to heat before death could be blisters on the skin, petechial hemorrhages in deeper skin layers and the conjunctivae [1,2]. It is important to mention that blisters may be present in postmortem burns as well, however they tend to be yellow, pale, filled with air and/or clear fluid with no red base present [3]. The presence of a line of redness around the burn has been regarded as evidence of vital reaction, however, it is not considered reliable as a red rim is frequently seen around postmortem burns [3]. In assessing antemortem signs of skin burns, the histopathology shows inflammatory tissue reaction: dilated capillaries, coagulative necrosis, swelling of epidermal cell nuclei, edema of the subepidermal connective tissue, vacuolization and elongation of cells and cell nuclei and polymorphonuclear leucocytic infiltration. These signs are not seen in the examination of postmortem burns [1,3].

Soot deposits in the respiratory tract are significant indicators of life in burnt bodies [4–6]. A living person (not necessarily a conscious one) will quickly inhale smoke and soot, which can not only settle in the above-mentioned organs, but can either cause their thermal burns [7]. Histological examination of the respiratory tract can demonstrate changes due thermal burns: hyperemia and edema of the mucosa in epiglottis, trachea and bronchi, vesicular detachment of the epithelium, pseudo-goblet cells and increased secretion of mucus are interpreted as signs of vital heat exposure if they occurred in combination [2]. After death, no significant amount of soot can pass through the voice chords or enter the trachea, which indicates that there will be no depositions found in postmortem burning cases [8,9]. It is important to mention, that there is a possibility that the integrity of the airways may have been disrupted in situations of severe burning and charring, which means soot may be present in air passages solely from exterior contamination and not from inhalation. The histological parameters should be likewise assessed only in context [7].

One of the most important tasks during an autopsy is the collection of fluid samples for toxicology testing. In addition to the usual toxicological examination (concentration of ethyl alcohol, presence/absence of narcotic substances in the blood), it is important to determine the amount of CO-Hb in the blood [4,8,9]. For nonsmokers <1 % and for smokers <10 % CO-Hb concentration in blood is considered normal. Greater values of CO-Hb confirms exposure to CO. When the CO-Hb saturation is 50% and more, CO poisoning is considered lethal, therefore this would support the hypothesis that the victim died in the fire. Lower levels of CO-Hb in the blood can suggest other causes of death, but even laboratory tests can sometimes be confusing. For instance, toxicity can be fatal at levels of 10% to 30% in people with underlying ischemic cardiomyopathy [10,11]. Moreover, in cases where combustion occurs very quickly, rapidly using up ambient oxygen (e.g., bushfires) the “flash fire” effect can cause death from lack of oxygen when CO levels are minimal. Therefore, the absence of CO in the peripheral blood in such circumstances should not be taken as evidence that death occurred before the fire started [7].

The combined effects of ethyl alcohol intoxication and CO poisoning are widely discussed by the authors. Alcohol at levels less than 0.3% protects against CO poisoning, although being drunk may have gotten the victim into the incident in the first place. CO toxicity is not due simply to the reduced oxygen-carrying capacity of the blood. A victim with 25% CO-Hb may die purely of CO.

Additionally, the high amount of CO-Hb determines the changed color of lividity. Usually, lividity is observed in bluish-purple discoloration of skin. Meanwhile, in large amount of CO-Hb, organs, muscles and skin are observed in cherry-red coloring [4,11]. Analyzing the blood’s cyanide and methemoglobin levels is one of the less common standard tests. This distinction is rarely ever used in practice and is only occasionally brought up in forensic literature [2].

Although autopsy is established as gold standard to determine cause of death, PMCT can be a valuable complement in the investigation of burnt bodies. In some cases, a criminal may attempt to conceal traumatic injuries by burning a body. One of the main challenges can be to distinguish if the injury is traumatic or the result of exposure to heat. PMCT, in addition to external, toxicological, and histological examination is reliable in assessing the primary cause of death, as it helps examine the body in more detail [12].

In highly charred bodies bones can be the only remaining organs to investigate. The thermal fractures present specific characteristics, which allow to differentiate them from traumatic fractures [13,14]. First, thermal bone injuries are found in the most superficial areas, while traumatic fractures depend on the characteristic of the trauma, therefore can include deep areas of the body. Flat bone heat fractures are longitudinal and trans-diploic with delamination and cortical network cracks while traumatic fractures are perpendicular to bone surface and touch the internal and external layers of compact cortical tissue. Opposed to traumatic fractures of the limbs, thermal fractures are frequently bilateral. Deep thermal bone lesions are rare in the projection of spine and pelvis because of protection by thick, soft tissues and are found on deeply charred bodies, therefore traumatic fractures are easily detected due to the remaining integrity of the adjacent soft tissue [14].

PMCT is advantageous in finding injuries in body areas which are not routinely dissected during autopsy (e.g., face), it excels in discovering subtle fractures which can be missed in heavily damaged areas during autopsy. PMCT can be a valuable tool to differentiate an epidural hematoma caused by blunt trauma from heat hematoma: unlike an epidural hematoma caused by trauma, a heat hematoma has a low radiodensity and arises regardless of sutural lines. The heat hematoma is crescent shaped and detached from the venous sinus, whereas an epidural hematoma is convex and lens shaped [4,15]. Moreover, measurement of CO-Hb can help to differentiate skull fracture caused by blunt trauma from heat hematoma, where CO-Hb level similar to that of the peripheral blood is detected [7]. Furthermore, PMCT can detect foreign bodies which can be of great use in victim identification (e.g., implants) or can help to uncover antemortem injuries (e.g., metal splinters).

However, some autopsy findings cannot be detected using PMCT: superficial thermal injuries and presence/absence of soot and thermal damage in the airways. As these findings are essential to prove vitality during the fire, PMCT cannot determine the cause of death, but it can be a valuable add-on in the postmortem investigation [4,16].

## Conclusions

When examining burnt and charred bodies, a complex and detailed assessment of all available data is required. During forensic investigation, it is important to use various diagnostic tools: although the main method is the autopsy, it must be remembered that additional diagnostic methods, including toxicological measurement of carboxyhemoglobin and postmortem computed tomography, can help to find out the real cause of death.

## References

[1] Chawla R, Chawla K, Sharma G, et al. Differentiation of antemortem & postmortem burns by histopathological examination. *Journal of Forensic Medicine and Toxicology*. 2014;31(2):70–74.

[2] Bohnert M, Werner CR, Pollak S. Problems associated with the diagnosis of vitality in burned bodies. *Forensic Sci Int.* 2003;135(3):197–205. doi: 10.1016/s0379-0738(03)00214-7 12927397

[3] Cooper PN. Burn Injury. In: Rutty GN, ed. *Essentials of Autopsy Practice*. Springer; 2006. doi:10.1007/1-84628-026-5_9

[4] Coty JB, Nedelcu C, Yahya S, Dupont V, Rougé-Maillart C, Verschoore M, et al. Burned bodies: post-mortem computed tomography, an essential tool for modern forensic medicine. *Insights Imaging*. 2018;9(5):731–743. doi:10.1007/s13244-018-0633-2 29882051 PMC6206378

[5] Fanton L, Jdeed K, Tilhet-Coartet S, Malicier D. Criminal burning. *Forensic Sci Int.* 2006;158(2–3):87–93. DOI:10.1016/j.forsciint.2005.04.040 15982840

[6] Kubo H, Hayashi T, Ago K, Ago M, Kanekura T, Ogata M. Forensic diagnosis of ante- and postmortem burn based on aquaporin-3 gene expression in the skin. *Leg Med (Tokyo)*. 2014;16(3):128–134. doi:10.1016/j.legalmed.2014.01.008 24508472

[7] Byard RW. The autopsy evaluation of “straightforward” fire deaths. *Forensic Sci Med Pathol*. 2018;14(3):273–275. doi:10.1007/s12024-017-9907-0 28831677

[8] Nikolić S, Živković V. Protrusion of the tongue in bodies burned after death: Two cases of arson to cover homicide. *Med Sci Law.* 2015;55(4):300–303. doi:10.1177/0025802414542260 25013164

[9] Tümer AR, Akçan R, Karacaoǧlu E, Balseven-Odabaşi A, Keten A, Kanburoǧlu Ç, et al. Postmortem burning of the corpses following homicide. *J Forensic Leg Med.* 2012;19(4):223–228. doi: 10.1016/j.jflm.2012.01.001 22520376

[10] Palmeri R, Gupta V. Carboxyhemoglobin Toxicity. In: *StatPearls*. Treasure Island (FL): StatPearls Publishing; April 21, 2022. 32491811

[11] Kinoshita H, Türkan H, Vucinic S, Naqvi S, Bedair R, Rezaee R, et al. Carbon monoxide poisoning. *Toxicol Reports*. 2020;7:169–173. doi:10.1016/j.toxrep.2020.01.005 PMC6992844 32015960

[12] Hueck U, Muggenthaler H, Hubig M, Heinrich A, Güttler F, Wagner R, et al. Forensic postmortem computed tomography in suspected unnatural adult deaths. *Eur J Radiol*. 2020;132:109297. doi: 10.1016/j.ejrad.2020.109297 33035918

[13] Galtés I, Scheirs S. Differentiation between perimortem trauma and heat-induced damage: the use of perimortem traits on burnt long bones. *Forensic Sci Med Pathol*. 2019;15(3):453–457. doi: 10.1007/s12024-019-00118-1 31098890

[14] Hammarlebiod S, Farrugia A, Bierry G, Raul JS, Willaume T. Thermal bone injuries: postmortem computed tomography findings in 25 cases. *Int J Legal Med.* 2022;136(1):219–227. doi:10.1007/s00414-021-02708-7 34570270

[15] Kawasumi Y, Usui A, Hosokai Y, Sato M, Funayama M. Heat haematoma: Post-mortem computed tomography findings. *Clin Radiol*. 2013;68(2):e95–e97. doi:10.1016/j.crad.2012.10.019 23219455

[16] de Bakker HM, Roelandt GHJ, Soerdjbalie-Maikoe V, van Rijn RR, de Bakker BS. The value of post-mortem computed tomography of burned victims in a forensic setting. *Eur Radiol*. 2019;29(4):1912–1921. doi: 10.1007/s00330-018-5731-5 30276675 PMC6420456

